# Real-World Effectiveness of 3 Types of Acellular Pertussis Vaccines Among Children Aged 3 Months–16 Years in Lu’an, China: A Matched Case-Control Study

**DOI:** 10.1093/ofid/ofaf082

**Published:** 2025-02-10

**Authors:** Wei Qin, Bingxin Ma, Huan Zhang, Yao Wang, Fan Pan, Yafei Chen, Yu Zhou, Yongyu Liu, Liguo Ma, Changjun Zhao, Yongjie Tian

**Affiliations:** Department of Epidemiology and Health Statistics, School of Public Health, Anhui Medical University, Hefei, Anhui, China; Department of Expanded Program on Immunization, Anhui Medical University Affiliated Lu’an Center for Disease Control and Prevention, Lu’an, Anhui, China; Department of Epidemiology and Health Statistics, School of Public Health, Anhui Medical University, Hefei, Anhui, China; Department of Expanded Program on Immunization, Anhui Medical University Affiliated Lu’an Center for Disease Control and Prevention, Lu’an, Anhui, China; Department of Expanded Program on Immunization, Anhui Medical University Affiliated Lu’an Center for Disease Control and Prevention, Lu’an, Anhui, China; Department of Expanded Program on Immunization, Anhui Medical University Affiliated Lu’an Center for Disease Control and Prevention, Lu’an, Anhui, China; Department of Expanded Program on Immunization, Anhui Medical University Affiliated Lu’an Center for Disease Control and Prevention, Lu’an, Anhui, China; Department of Expanded Program on Immunization, Jinzhai County Center for Disease Control and Prevention, Lu’an, Anhui, China; Department of Expanded Program on Immunization, Jin’an District Center for Disease Control and Prevention, Lu’an, Anhui, China; Department of Expanded Program on Immunization, Anhui Medical University Affiliated Lu’an Center for Disease Control and Prevention, Lu’an, Anhui, China; Department of Expanded Program on Immunization, Jin’an District Center for Disease Control and Prevention, Lu’an, Anhui, China; Public Health Department, Lu’an Hospital of Anhui Medical University, Lu’an, Anhui, China

**Keywords:** acellular pertussis vaccine, case-control study, pertussis, vaccine effectiveness, whooping cough

## Abstract

**Background:**

The real-world vaccine effectiveness (VE) of the diphtheria, tetanus, and acellular pertussis (DTaP), DTaP–*Haemophilus influenzae* type b (Hib), and DTaP–inactivated polio (IPV)/Hib vaccines has not been thoroughly evaluated in China. Additionally, there are limited data on the VE of acellular pertussis–containing vaccines (aPVs) when used interchangeably.

**Methods:**

We conducted a matched case-control study to estimate the VE of aPVs against polymerase chain reaction–confirmed pertussis infection in Lu’an in 2024. A conditional logistic regression model was used to compare the odds ratios (ORs) of vaccination between cases and controls. VE was calculated as [(1 – adjusted OR) × 100%], and 95% confidence intervals (CIs) were computed around the estimates.

**Results:**

A total of 1936 children aged 3 months to 16 years were included in the study. The overall VE was 77.3% (95% CI, 35.2%–92.1%). The VE for fully vaccinated children was 88.4% (95% CI, 57.3%–96.8%), while the VE for partially vaccinated children was 77.4% (95% CI, 35.5%–92.1%). The VE of DTaP, DTaP-Hib, and DTaP-IPV/Hib was 75.8% (95% CI, 29.7%–91.7%), 83.2% (95% CI, 47.8%–94.6%), and 79.8% (95% CI, 36.5%–93.6%), respectively. Compared with mixed vaccination (65.3%.), the incremental VE of DTaP, DTaP-Hib, and DTaP-IPV/Hib was 31.0% (95% CI, 1.0%–51.9%), 52.9% (95% CI, 19.1%–72.6%), and 41.1% (95% CI, −18.7% to 71.8%), respectively. We observed a decline in VE over time, decreasing from 76.5% (95% CI, 33.0%–91.7%) within the first 2 years to −5.5% (95% CI, −495.2% to 81.3%) after 6 years or more.

**Conclusions:**

All aPVs provide significant protection against pertussis infection, although this protection wanes over time. The VE appears to decrease materially if these vaccines are administered alternately in an individual's routine immunization schedule.

Pertussis is a highly contagious acute respiratory disease caused by the bacterium *Bordetella pertussis*. While individuals of all age groups are susceptible to this infection, it can be particularly fatal for unvaccinated individuals, especially very young infants [1, 2]. The widespread use of the pertussis vaccine has dramatically decreased the disease burden, maintaining it at a low level for many years. However, despite high vaccination coverage in recent decades, many countries have reported a resurgence of pertussis [3–5]. Belatedly but unsurprisingly, this resurgence has also been observed in China [6]. Data released by the National Health Commission of China showed that reported pertussis cases have risen sharply since January 2024, with reported cases reaching up to 15 275 in January, 27 078 in March, and rising further to 97 669 in May [7, 8]. This increase in pertussis cases may shake parents' confidence in the vaccine and lead to parental vaccine hesitancy, which in turn may increase the risk of other vaccine-preventable infectious diseases in children [9]. Hence, it is crucial to promptly assess the vaccine effectiveness (VE) of authorized pertussis vaccines in China.

Pertussis vaccines are available in 2 formulations: whole-cell pertussis (wP) and acellular pertussis (aP). These vaccines are often included in combination vaccines, such as the diphtheria, tetanus, and whole-cell pertussis vaccine (DTwP) and the diphtheria, tetanus, and acellular pertussis combined vaccine (DTaP) [10]. According to the different extraction processes of pertussis antigen components, the aP vaccine can be further classified into 2 categories: component-purified aP vaccine and co-purified aP vaccine [11]. Evidence from previous studies has suggested that receipt of any pertussis vaccine confers protection against pertussis disease, although this protection wanes rapidly [12–15]. Sánchez-González et al estimated that the VE of the first 3 doses of wP-containing vaccine (wPV) and aP-containing vaccine (aPV) against notified pertussis was as high as 96.4% and 95.7%, respectively [15]. Wilkinson et al reported that the VE of aPV was 85%, while the VE of wPV was only 35% [14]. Many factors, such as differences in study design methodologies and variations in vaccine types used across countries, contribute to these variable results [16]. In China, the DTwP was included in the Expanded Program on Immunization (EPI) in 1978, and it was gradually replaced by the DTaP from 2007 to 2013 [11, 17]. Three types of aPVs have been authorized and widely used in China over the last 10 years, including the diphtheria, tetanus, and acellular pertussis vaccine (DTaP); the diphtheria, tetanus, acellular pertussis, and *Haemophilus influenzae* type b combined vaccine (DTaP-Hib); and the diphtheria, tetanus, pertussis (acellular, component), poliomyelitis (inactivated) vaccine, and *Haemophilus influenzae* type b conjugate vaccine, absorbed (DTaP-IPV/Hib) [18]. Among these, DTaP and DTaP-Hib are domestic vaccines classified as co-purified aPVs, while DTaP-IPV/Hib is an imported vaccine produced by Sanofi Pasteur and is classified as a component-purified aPV. A few studies have reported the safety and effectiveness of DTaP and aPV in China [11, 18–21]. Zhu et al found that the DTaP VEs for 1–4 doses in children aged ≤2 years were 24.1%, 45.5%, 57.9%, and 87.1%, respectively [11]. A matched case-control study conducted in Shenzhen, China, revealed that the overall VE of aPVs against pertussis was 90.75% in children aged 4 months–6 years [19]. However, there are limited real-world VE data on DTaP-Hib and DTaP-IPV/Hib. Specifically, there are almost no available data regarding the effectiveness of these vaccines when used interchangeably.

In this study, we conducted a 1:3 age-matched case-control study to evaluate the real-world effectiveness of 3 types of aPVs in China. We believe that this study could address the evidence gap and quantify the real-world VE of DTaP, DTaP-Hib, and DTaP-IPV/Hib vaccines used in China.

## METHODS

### Study Setting

**Figure 1.**
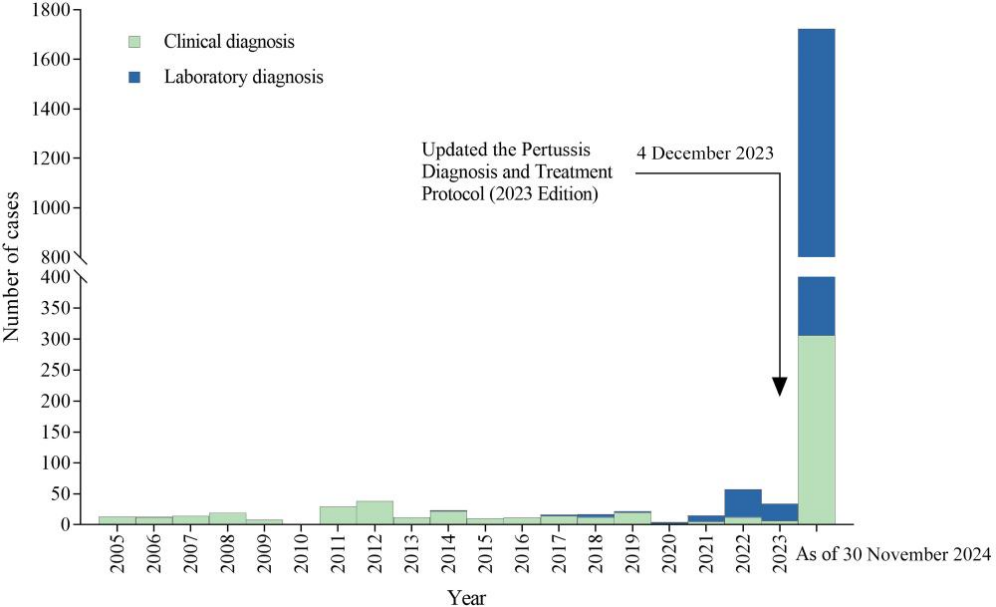
Number of pertussis cases by method of diagnosis in Lu’an, China, 1 January 2005 through 30 November 2024.

According to the Chinese Information System for Disease Control and Prevention, between 1 January and 30 November 2024, a total of 1723 pertussis cases were reported in Lu’an, comprising 306 clinically diagnosed cases and 1417 laboratory-confirmed cases. This figure represents 5 times the total number of reported cases over the past 19 years (2005–2023) and is 50 times greater than the number reported last year (Figure 1). We also observed that 47.1% (668/1417) of the laboratory-confirmed cases were reported by Lu’an Hospital of Anhui Medical University (hospital 1), Jinzhai County People's Hospital (hospital 2), and Jin’an Maternity and Child Health Care Hospital (hospital 3) after testing positive by real-time polymerase chain reaction (PCR) for the detection of *Bordetella pertussis.* The pertussis PCR assay kits used in hospital 1 and hospital 2 were manufactured by BioGerm Medical Technology Co, Ltd, targeting the IS1633 gene. The pertussis PCR assay kits used in hospital 3 were manufactured by Sansure Biotech Inc, targeting the IS481 gene. Both of these kits have been approved by the National Medical Products Administration to confirm cases of pertussis. Additionally, the procedures and preparation of consumables for the PCR assay were conducted in accordance with the manufacturer's instruction manual. We consider the pertussis test results from these 3 hospitals to be reliable. Therefore, we conducted a 1:3 age-matched case-control study in the above 3 public hospitals to explore the effectiveness of aPVs against pertussis infection.

### Study Participants

**Figure 2.**
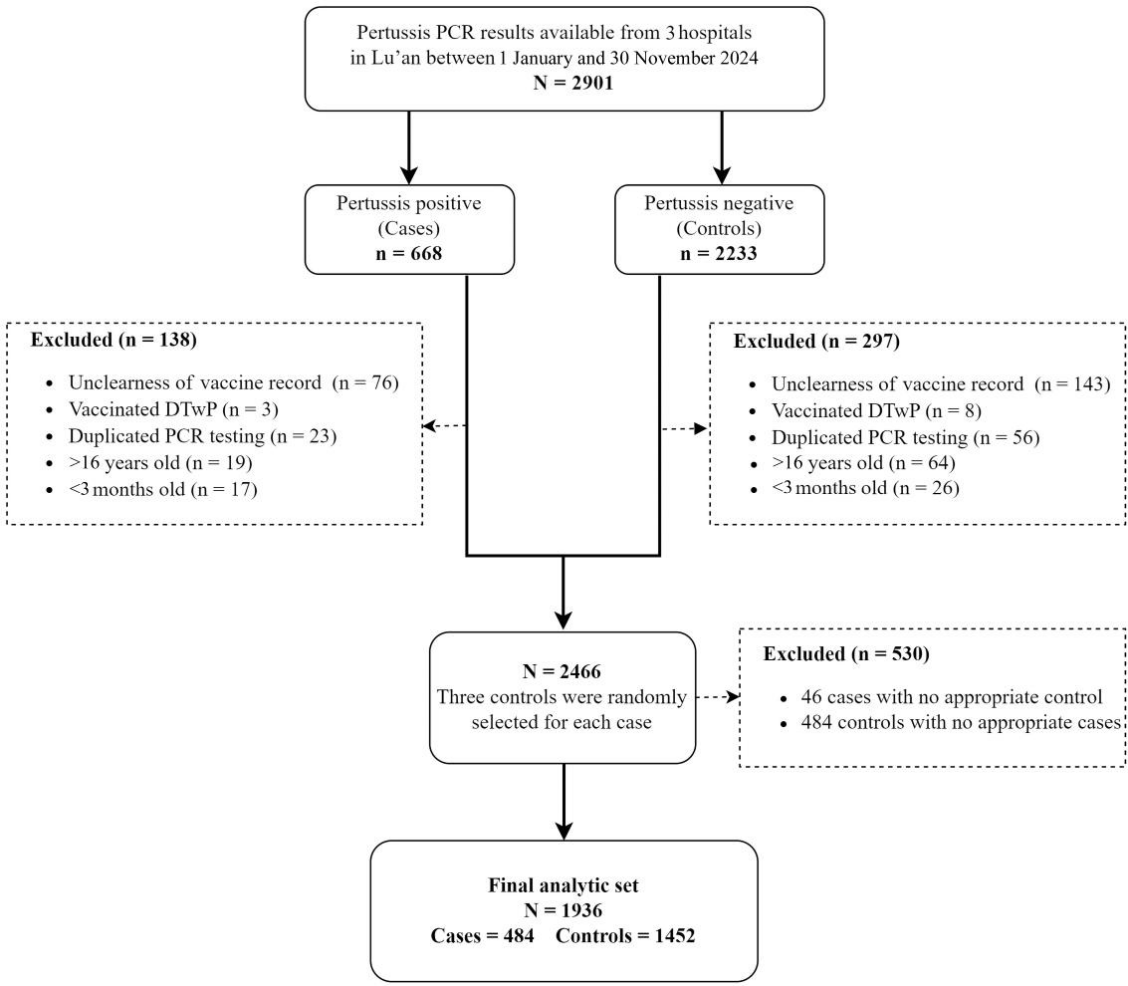
Flowchart for study population. Abbreviations: DTwP, diphtheria, tetanus, and whole-cell pertussis combined vaccine; PCR, real-time polymerase chain reaction.

Eligible participants were children aged 3 months to 16 years at admission who presented with symptoms resembling pertussis prior to PCR testing. We chose this age group to ensure that all study participants (both cases and controls) had the opportunity to receive the aPVs. Their clinical symptoms needed to meet the diagnostic criteria outlined in the Pertussis Diagnosis and Treatment Protocol (2023 edition), which includes a paroxysmal spasmodic cough lasting at least 2 weeks, an inspiratory whoop following coughing, or posttussive vomiting. The inclusion criteria for cases are as follows: (1) tested positive for pertussis via PCR; (2) aged between 3 months and 16 years; and (3) with clear immunization records. We randomly selected 3 controls for each case during the study period. The inclusion criteria for controls are as follows: (1) tested negative for pertussis via PCR; (2) aged between 3 months and 16 years; (3) with clear immunization records; (4) controls selected for cases aged 3–4 months were within a 2-month age group of the case; (5) controls selected for cases aged 5–18 months were within 5–18 months old; (6) controls selected for cases aged ≥18 months were within a 2-year age group of the case; and (7) control cases should be sourced from the same hospital as the selected cases. Participants were excluded if they lacked laboratory results, were >16 years of age or <3 months of age, had missing immunization records, had received any dose of DTwP, or had undergone repeated PCR testing. In total, 484 cases and 1452 controls from 3 public hospitals were enrolled in the matched case-control study (Figure 2).

### Vaccination History

We obtained vaccination data and critical demographic information from the Anhui Immunization Information Management System (AIIMS). The AIIMS is an internet-based management platform that maintains essential demographic information and immunization records for vaccine recipients, as detailed in our previous study [22, 23]. The aPVs in China adhere to a complete vaccination schedule that consists of 4 doses: DTaP and DTaP-Hib at 3, 4, 5, and 18 months of age, and DTaP-IPV/Hib at 2, 3, 4, and 18 months of age. A valid dose was defined as the corresponding dose administered >2 weeks before the onset of illness in cases or PCR testing in controls [13, 24]. Participants who received all 4 doses of aPVs during the study period were classified as fully vaccinated. Those who received only 1–3 doses of aPVs were classified as partially vaccinated. Mixed vaccination was defined as the administration of DTaP, DTaP-Hib, and DTaP-IPV/Hib alternately within an individual's routine immunization schedule. The Lu’an Municipal Centers for Disease Control and Prevention staff reviewed and confirmed all vaccination status and timing data.

### Ethics Statement

Emergency surveillance and field investigation of a notifiable infectious disease are part of the public health response, and ethical clearance was not required. All authors and participants will keep all data in this study confidential.

### Statistical Analysis

All collected data were entered into Microsoft Excel 2019 software for data cleaning. Differences in variables between the exposure group and the control group were assessed using the χ^2^ test or the Mann-Whitney *U* test, as appropriate. A conditional logistic regression model was employed to estimate the odds ratio (OR) and the 95% confidence interval (CI) for test-positive cases compared to test-negative controls, while adjusting for age, sex, and hospital factors. VE was calculated as [(1 – adjusted OR) × 100%]. Incremental VE of 3 types of aPVs compared with mixed vaccination was estimated by using the same equation. Statistical analyses were performed using IBM SPSS Statistics for Windows version 20.0 (IBM Corp, Armonk, New York). Two-sided *P* values were considered statistically significant at <.05.

### Sensitivity Analysis

We conducted a sensitivity analysis for each subgroup by applying the final conditional logistic regression model to a redefined valid dose. In the sensitivity analyses, a valid dose was defined as the corresponding dose administered >1 week before the onset of illness in cases and before PCR testing in controls.

## RESULTS

### Baseline Characteristics

During the study period, we identified 1936 participants, including 484 PCR-positive cases and 1452 PCR-negative controls. Table 1 presents the baseline characteristics of all participants. We found that the median time interval since the last dose of aPVs for cases was longer than that of controls (4.3 years vs 3.8 years, *P* = .009). The other characteristics were similar between the 2 groups.

**Table 1.** Baseline Characteristics of the Study Participants

Characteristic	Total (n = 1936)	Cases (n = 484)	Controls (n = 1452)	*P* Value^a^
Hospital				
Hospital 1	1620 (83.7)	405 (83.7)	1215 (83.7)	1.000^b^
Hospital 2	184 (9.5)	46 (9.5)	138 (9.5)	
Hospital 3	132 (6.8)	33 (6.8)	99 (6.8)	
Sex				
Male	1070 (55.3)	271 (56.0)	799 (55.0)	.712^b^
Female	866 (44.7)	213 (44.0)	653 (45.0)	
Age, y, median (IQR)	5.5 (4.2–7.4)	5.8 (4.5–7.3)	5.4 (4.2–7.4)	.096^c^
Vaccination status				
Unvaccinated	33 (1.7)	13 (2.7)	20 (1.4)	.155^b^
Partial vaccination	213 (11.0)	52 (10.7)	161 (11.1)	
Full vaccination	1690 (87.3)	419 (86.6)	1271 (87.5)	
Vaccine type^d^				
DTaP	1487 (78.1)	371 (78.8)	1116 (77.9)	.067^b^
DTaP-Hib	156 (8.2)	29 (6.2)	127 (8.9)	
DTaP-IPV/Hib	75 (3.9)	15 (3.2)	60 (4.2)	
Mixed vaccination	185 (9.7)	56 (11.9)	129 (9.0)	
Time since the last dose vaccination^d^, y, median (IQR)	3.9 (2.5–5.7)	4.3 (2.9–5.8)	3.8 (2.5–5.7)	.009^c^

Data are presented as No. (%) unless otherwise indicated.

Abbreviations: DTaP, diphtheria, tetanus, and acellular pertussis combined vaccine; DTaP-Hib, diphtheria, tetanus, acellular pertussis, and *Haemophilus influenzae* type b combined vaccine; DTaP-IPV/Hib, diphtheria, tetanus, pertussis (acellular, component), poliomyelitis (inactivated) vaccine, and *Haemophilus influenzae* type b conjugate vaccine, absorbed; IQR, interquartile range.

^a^Cases versus controls.

^b^χ^2^ test.

^c^Independent-samples Mann-Whitney *U* test.

^d^Unvaccinated participants were not included.

### Vaccine Effectiveness

Table 2 shows crude ORs, adjusted ORs, and adjusted VEs in different subgroups. A total of 471 (97.3%) cases and 1432 (98.6%) controls received at least 1 dose of aPVs. The overall adjusted VE for children aged 3 months to 16 years was 77.3% (95% CI, 35.2%–92.1%). The adjusted VE for fully vaccinated children was 88.4% (95% CI, 57.3%–96.8%), while for those with partial vaccination, it was 77.4% (95% CI, 35.5%–92.1%). The overall adjusted VE of DTaP, DTaP-Hib, and DTaP-IPV/Hib was 75.8% (95% CI, 29.7%–91.7%), 83.2% (95% CI, 47.8%–94.6%), and 79.8% (95% CI, 36.5%–93.6%), respectively. Notably, the overall adjusted VE for children with mixed vaccination was only 65.3% (95% CI, −5.7% to 88.6%), which is lower than the VEs observed when exclusively using the same type of aPVs during the vaccination course. Compared with mixed vaccination, we observed that the incremental VE of DTaP, DTaP-Hib, and DTaP-IPV/Hib was 31.0% (95% CI, 1.0%–51.9%), 52.9% (95% CI, 19.1%–72.6%), and 41.1% (95% CI, −18.7% to 71.8%), respectively. We did not observe a statistically significant difference in incremental VE for DTaP-Hib and DTaP-IPV/Hib when compared to DTaP (*P* > .05). Subgroup analyses by time since the last dose of aPV vaccination showed a declining trend in overall VE, with an adjusted VE of 76.5% (95% CI, 33.0%–91.7%) within the first 2 years, 74.3% (95% CI, 4.2%–93.1%) at 2–3 years, −113.3% (95% CI, −957.5% to 57.0%) at 4–5 years, and −5.5% (95% CI, −495.2% to 81.3%) at 6 years or more.

**Table 2.** Crude Odds Ratios, Adjusted Odds Ratios, and Adjusted Vaccine Effectiveness of Acellular Pertussis–Containing Vaccines in the Matched Case-Control Study

Group	Cases, No. (%)	Controls, No. (%)	Crude OR (95% CI)	Adjusted OR (95% CI)	Adjusted VE, % (95% CI)
Overall					
Unvaccinated	13 (2.7)	20 (1.4)	Ref	Ref	Ref
With any vaccine	471 (97.3)	1432 (98.6)	0.299 (.107–.836)	0.227 (.079–.648)	**77.3 (35.2–92.1)**
By vaccination course					
Unvaccinated	13 (2.7)	20 (1.4)	Ref	Ref	Ref
Partial vaccination	52 (10.7)	161 (11.1)	0.299 (.107–.836)	0.226 (.079–.645)	**77.4 (35.5–92.1)**
Full vaccination	419 (86.6)	1271 (87.5)	0.179 (.051–.635)	0.116 (.032–.427)	**88.4 (57.3–96.8)**
By vaccine type					
Unvaccinated	13 (2.7)	20 (1.4)	Ref	Ref	Ref
DTaP	371 (76.7)	1116 (76.9)	0.327 (.116–.926)	0.242 (.083–.703)	**75.8 (29.7–91.7)**
DTaP-Hib	29 (6.0)	127 (8.7)	0.217 (.071–.658)	0.168 (.054–.522)	**83.2 (47.8–94.6)**
DTaP-IPV/Hib	15 (3.1)	60 (4.1)	0.249 (.081–.768)	0.202 (.064–.635)	**79.8 (36.5–93.6)**
Mixed vaccination	56 (11.6)	129 (8.9)	0.433 (.146–1.288)	0.347 (.114–1.057)	65.3 (−5.7 to 88.6)
By time since vaccination, y					
Unvaccinated	13 (2.7)	20 (1.4)	Ref	Ref	Ref
0–1	86 (17.8)	289 (19.9)	0.299 (.107–.836)	0.235 (.083–.670)	**76.5 (33.0–91.7)**
2–3	125 (25.8)	464 (32.0)	0.466 (.128–1.695)	0.257 (.069–.958)	**74.3 (4.2–93.1)**
4–5	161 (33.3)	370 (25.5)	6.721 (1.419–31.835)	2.133 (.430–10.575)	−113.3 (−957.5 to 57.0)
≥6	99 (20.5)	309 (21.3)	4.860 (.924–25.563)	1.055 (.187–5.952)	−5.5 (−495.2 to 81.3)

Values in bold denote significant VE estimates.

Abbreviations: CI, confidence interval; DTaP, diphtheria, tetanus, and acellular pertussis combined vaccine; DTaP-Hib, diphtheria, tetanus, acellular pertussis, and *Haemophilus influenzae* type b combined vaccine; DTaP-IPV/Hib, diphtheria, tetanus, pertussis (acellular, component), poliomyelitis (inactivated) vaccine, and *Haemophilus influenzae* type b conjugate vaccine, absorbed; OR, odds ratio; Ref, reference; VE, vaccine effectiveness.

### Sensitivity Analyses

Restricting the valid dosage to those administered >1 week before the onset of illness in cases and before PCR testing in controls did not significantly alter the adjusted VEs in each subgroup (Table 3). The overall adjusted VE in the sensitivity analysis was 74.2% (95% CI, 24.6%–91.2%), compared to 77.4% (95% CI, 35.2%–92.1%) for the results of the original analysis. Similar robust results were also observed in the sensitivity analysis for the other subgroups.

**Table 3.** Sensitivity Analysis for the Crude Odds Ratios, Adjusted Odds Ratios, and Vaccine Effectiveness of Acellular Pertussis–Containing Vaccines in the Matched Case-Control Study

Groups	Cases, No. (%)	Controls, No. (%)	Crude OR (95% CI)	Adjusted OR (95% CI)	Adjusted VE, % (95% CI)
Overall					
Unvaccinated	12 (2.5)	20 (1.4)	Ref	Ref	Ref
With any vaccine	472 (97.5)	1432 (98.6)	0.299 (.107–.836)	0.258 (.088–.754)	**74.2 (24.6–91.2)**
By vaccination course					
Unvaccinated	12 (2.5)	20 (1.4)	Ref	Ref	Ref
Partial vaccination	53 (11.0)	159 (11.0)	0.343 (.120–.977)	0.256 (.088–.750)	**74.4 (25.0–91.2)**
Full vaccination	419 (86.6)	1273 (87.7)	0.193 (.054–.689)	0.123 (.033–.458)	**87.7 (54.2–96.7)**
By vaccine type					
Unvaccinated	12 (2.5)	20 (1.4)	Ref	Ref	Ref
DTaP	371 (76.7)	1115 (76.8)	0.373 (.130–1.070)	0.275 (.093–.812)	**72.5 (18.8–90.7)**
DTaP-Hib	30 (6.2)	127 (8.7)	0.254 (.083–.783)	0.197 (.063–.621)	**80.3 (37.9–93.7)**
DTaP-IPV/Hib	15 (3.1)	60 (4.1)	0.274 (.086–.872)	0.220 (.067–.717)	**78.0 (28.3–93.3)**
Mixed vaccination	56 (11.6)	130 (9.0)	0.488 (.162–1.474)	0.389 (.125–1.204)	61.1 (−20.4 to 87.5)
By time since vaccination, y					
Unvaccinated	12 (2.5)	20 (1.4)	Ref	Ref	Ref
0–1	87 (18.0)	289 (19.9)	0.343 (.120–.977)	0.268 (.092–.780)	**73.2 (22.0–90.8)**
2–3	125 (25.8)	464 (32.0)	0.534 (.145–1.973)	0.292 (.077–1.112)	**70.8 (−11.2 to 92.3)**
4–5	161 (33.3)	370 (25.5)	7.708 (1.607–36.964)	2.436 (.484–12.257)	−143.6 (−1125.7 to 51.6)
≥6	99 (20.5)	309 (21.3)	5.574 (1.047–29.659)	1.207 (.211–6.903)	−20.7 (−590.3 to 78.9)

Values in bold denote significant VE estimates.

Abbreviations: CI, confidence interval; DTaP, diphtheria, tetanus, and acellular pertussis combined vaccine; DTaP-Hib, diphtheria, tetanus, acellular pertussis, and *Haemophilus influenzae* type b combined vaccine; DTaP-IPV/Hib, diphtheria, tetanus, pertussis (acellular, component), poliomyelitis (inactivated) vaccine, and *Haemophilus influenzae* type b conjugate vaccine, absorbed; OR, odds ratio; Ref, reference; VE, vaccine effectiveness.

## DISCUSSION

Our study estimated that the overall VE of aPVs for children aged 3 months to 16 years against PCR-confirmed pertussis infection was moderate, at approximately 77.3%. The VE for fully vaccinated children was 88.4%, while the VE for partially vaccinated children was 77.4%. We also found that the overall VE for children with mixed vaccination was only 65.3%, which is lower than the VEs observed when exclusively using the same type of aPVs throughout the vaccination course. Furthermore, we observed a decline in overall VE over time, decreasing from 76.5% (95% CI, 33.0%–91.7%) within the first 2 years to −5.5% (95% CI, −495.2% to 81.3%) after 6 years or more.

Despite the implementation of the pertussis vaccine in the universal immunization program, the recurrence of pertussis has been observed in recent years in China and other regions. This resurgence of pertussis can be attributed to a complex interplay of factors, including at least vaccine types, vaccination rates, waning immunity, pathogen evolution, and surveillance efforts [25, 26]. Among these factors, the effectiveness and long-term immune persistence of the currently used pertussis vaccines are the most significant concerns for public health policy-makers. Evidence from previous studies has shown that the receipt of both aPV and wPV confers protection against disease, but the VE of aPV appears to be lower than that of wPV, and this protection wanes rapidly for aPV [10, 27]. We found that the overall VE of the aPVs observed in this study was slightly lower than the results reported in previous studies conducted in China [11, 19, 20] and other countries [12, 14, 28, 29]. This discrepancy can largely be attributed to the age range of our study participants (ranging from 3 months to 16 years), all of whom were born after China implemented the EPI in 2007. We also observed that the overall VE of aPVs for children aged 3 months to 16 years was 77.3%, but the VE declined from 76.5% within the first 2 years to −5.5% after 6 years or more. These findings suggest that the current immunization program in China needs optimization, including the consideration of an additional booster dose at an appropriate time for older children and adolescents. However, Crowcroft et al suggested that caution should be exercised by researchers and decision-makers when evaluating evidence on the optimal timing of boosters [16]. They considered that the duration of protection from pertussis vaccines is unclear because estimates vary by study design. They observed lower VE and faster waning in the test-negative design (TND) study than in the frequency-matched design (FMD) case-control study. Previous studies employing TNDs to evaluate pertussis vaccine efficacy highlighted similar considerations and utilized various methods to minimize bias [30–32]. Considering that Crowcroft et al concluded that FMD more effectively adjusted healthcare-seeking behavior than TND [16], we employed an age-matched case-control study to estimate the VE in the present study. However, we also noticed that this design may have led to an overestimation of the overall VE, as a significant proportion of children in the control group were vaccinated with aPVs (98.6%).

We also conducted subgroup analyses to evaluate the overall VE of DTaP, DTaP-Hib, and DTaP-IPV/Hib, both individually and when used interchangeably. The aPVs usually contain 1 or more of the following purified antigens: pertussis toxoid, filamentous hemagglutinin, pertactin, and fimbriae types 2 and 3 [2, 4]. Although existing studies have shown that multicomponent aPVs provide higher protective efficacy compared to 1-component and 2-component aPVs against both typical whooping cough and mild pertussis disease [33, 34], the World Health Organization (WHO) suggests that it still needs to be interpreted with caution. Although no statistically significant differences were identified due to the sample size, the VE point estimates were slightly higher for DTaP-Hib (83.2%) and DTaP-IPV/Hib (79.8%) compared to DTaP (75.8%). This result also warrants our attention, and future studies should expand the sample size to accurately evaluate the differences in the VE of the 3 types of aPVs in real-world settings. In addition, the WHO and the United States Advisory Committee on Immunization Practices recommend using the same type of aPVs for all doses of the vaccination series whenever possible [2, 4], and our findings strongly support this recommendation. We observed that the VE appears to decrease materially if these vaccines are administered alternately in an individual's routine immunization schedule. Our study showed that the adjusted VE of mixed vaccination was only 65.3% (95% CI, −5.7% to 88.6%), which is lower than the VEs observed when exclusively using the same type of aPVs during the vaccination course. We observed that the incremental VE of DTaP (31.0%), DTaP-Hib (52.9%), and DTaP-IPV/Hib (41.1%) was substantial compared to mixed vaccination. Therefore, we also recommend avoiding interchangeably using aPVs unless the previous type is unknown or unavailable.

One strength of this study is that we quantitatively assessed the real-world effectiveness of all 3 types of aPVs licensed in China, both when used individually and when used interchangeably. However, our findings are subject to several limitations. First, control children are more likely to be vaccinated due to the high vaccination rate of aPVs in China, especially when multiple controls are selected for statistical efficacy, which may lead to an overestimation of VE. Additionally, the sensitivity and specificity of the PCR tests may have influenced the determination of case or control status. Nevertheless, all pertussis PCR assay kits utilized in the 3 selected hospitals are approved by the National Medical Products Administration for confirming cases of pertussis. Furthermore, all participants included in this study were required to exhibit pertussis-like symptoms by the diagnostic criteria outlined in the Pertussis Diagnosis and Treatment Protocol (2023 edition) or to have an epidemiological association with a confirmed case of pertussis prior to undergoing PCR testing. These measures significantly reduce the risk of misclassification associated with the TND. Third, we lacked information on the clinical characteristics and hospitalization status of participants, which could have provided additional insights into the VEs of different types of aPVs. Finally, we did not analyze the VE of the aPVs in each age group, as the limited number of unvaccinated children in this study precluded a subgroup analysis. In the future, we will continue to conduct prospective cohort studies to provide further evidence regarding the real-world effectiveness of the aPVs available in China.

In summary, all 3 types of aPVs licensed in China offer substantial protection against pertussis infection, although this protection wanes rapidly. Furthermore, we found that the VE was significantly lower for mixed vaccination regimens, indicating a preference for administering the same type of aPV for all doses in the vaccination series whenever possible. Our findings provide further evidence for public health policy-makers to enhance and implement effective pertussis immunization strategies in China.

## Notes

***Acknowledgments.*** We gratefully acknowledge the contributions of the staff from Lu’an Hospital of Anhui Medical University and Jinzhai County People's Hospital. We also thank the Centers for Disease Control and Prevention staff from Shucheng, Jinzhai, Yeji, Jin’an, Yu’an, Huoshan, and Huoqiu for their assistance in collecting and integrating the vaccination data.

***Author contributions.*** W. Q. designed this study and drafted the manuscript. H. Z. and B. M. participated in the data cleaning and analysis. Y. T., Y. L., C. Z., and Y. Z. provide and responsible for collecting and reviewing the pertussis PCR test results from the hospital's visiting population. Y. C., L. M., Y. W., and F. P. participated in the immunization record review. All the authors reviewed and edited the final manuscript.

***Financial support***. This work was supported by the Summit Discipline Construction Project of the School of Public Health at Anhui Medical University (Collaborative Education Innovation Program) in 2024 (grant number 2024GWXTYRY003); the Scientific Research Project of the Health Commission of Anhui Province (grant number AHWJ2022c040); and the Special Funds for the Expanded Program on Immunization in 2024 from the Lu’an Municipal Government.
